# HTSstation: A Web Application and Open-Access Libraries for High-Throughput Sequencing Data Analysis

**DOI:** 10.1371/journal.pone.0085879

**Published:** 2014-01-27

**Authors:** Fabrice P. A. David, Julien Delafontaine, Solenne Carat, Frederick J. Ross, Gregory Lefebvre, Yohan Jarosz, Lucas Sinclair, Daan Noordermeer, Jacques Rougemont, Marion Leleu

**Affiliations:** 1 School of Life Sciences, Ecole Polytechnique Fédérale de Lausanne (EPFL), Lausanne, Switzerland; 2 Swiss Institute of Bioinformatics (SIB), Lausanne, Switzerland; 3 Swiss Institute for Experimental Cancer Research (ISREC), Lausanne, Switzerland; Université Paris-Sud, France

## Abstract

The HTSstation analysis portal is a suite of simple web forms coupled to modular analysis pipelines for various applications of High-Throughput Sequencing including ChIP-seq, RNA-seq, 4C-seq and re-sequencing. HTSstation offers biologists the possibility to rapidly investigate their HTS data using an intuitive web application with heuristically pre-defined parameters. A number of open-source software components have been implemented and can be used to build, configure and run HTS analysis pipelines reactively. Besides, our programming framework empowers developers with the possibility to design their own workflows and integrate additional third-party software. The HTSstation web application is accessible at http://htsstation.epfl.ch.

## Introduction

### Background

High-throughput sequencing (HTS) has produced a major shift in the amount and speed of data production in genomics. More importantly, the range of applications of the technology is constantly expanding. The importance of bioinformatics has grown in parallel and with it the necessity to implement efficient and reproducible analysis processes specifically adapted to each particular application. It is also of prime importance to allocate bioinformaticians' efforts and time to addressing the specific scientific questions of each research project and therefore to package the proven and tested parts of each analysis into convenient software. Besides, the academic world generates a wealth of freely available software and this must be exploited for the sake of efficiency without preconceptions of programming language, platform or style.

In this context, we have undertaken to implement an analysis platform that helps us flexibly introduce our own innovations side-by-side with publicly available tools and make them accessible to the scientific community. The work we present in this paper grew out of our first-hand experience with analyzing HTS data within scientific collaborations [1]–[11] where fast and biologically convincing analysis results were the measure of success. We quickly realized, though, that providing a stable, more generic implementation of the methods used for these projects was essential to keep up with the flow of data. HTSstation was developed in this perspective.

While similar to other web-based applications (e.g. Chipster [12], GeneProf [13], Galaxy [14]), HTSstation distinguishes itself in a number of ways:

- The job definition includes the experimental design (biological conditions and replicates) with intuitive joining or splitting of sample groups at any step of the workflow.
- Result files are named and displayed according to the experimental design matrix, with links to their original source, to minimize risks of sample mix-up.
- Visualization of the data is externalized (for example to the UCSC genome browser [15]), but facilitated by providing prepared BED files grouping results by job, by type or by condition. Similarly data can be shared via obfuscated URLs.
- Reproducibility and tracking of analyses is facilitated by a job cloning capability and single click transfer of files from one pipeline to another.
- Full customization of the pipeline is available but at clearly separated levels that coincide with different user experiences: preconfigured workflows require minimal user-defined options, extensive customization of individual jobs is available via structured configuration files, and a programming framework is offered to reconfigure and extend entire workflows.
- The web-based client-server architecture was designed not to require any specific resource or software from the client in spite of the large (and increasing) size of files (FASTQ, BAM, genome indexes) and the concurrent computational burden. In particular, large files are not uploaded via the web interface, but pulled by the server from a public location.
- The back-end is fully adapted to a High Performance Computing (HPC) environment with built-in parallelization of tasks, clear separation between temporary buffer storage and ultimate archival of analysis results.

### Design

**Figure 1:**
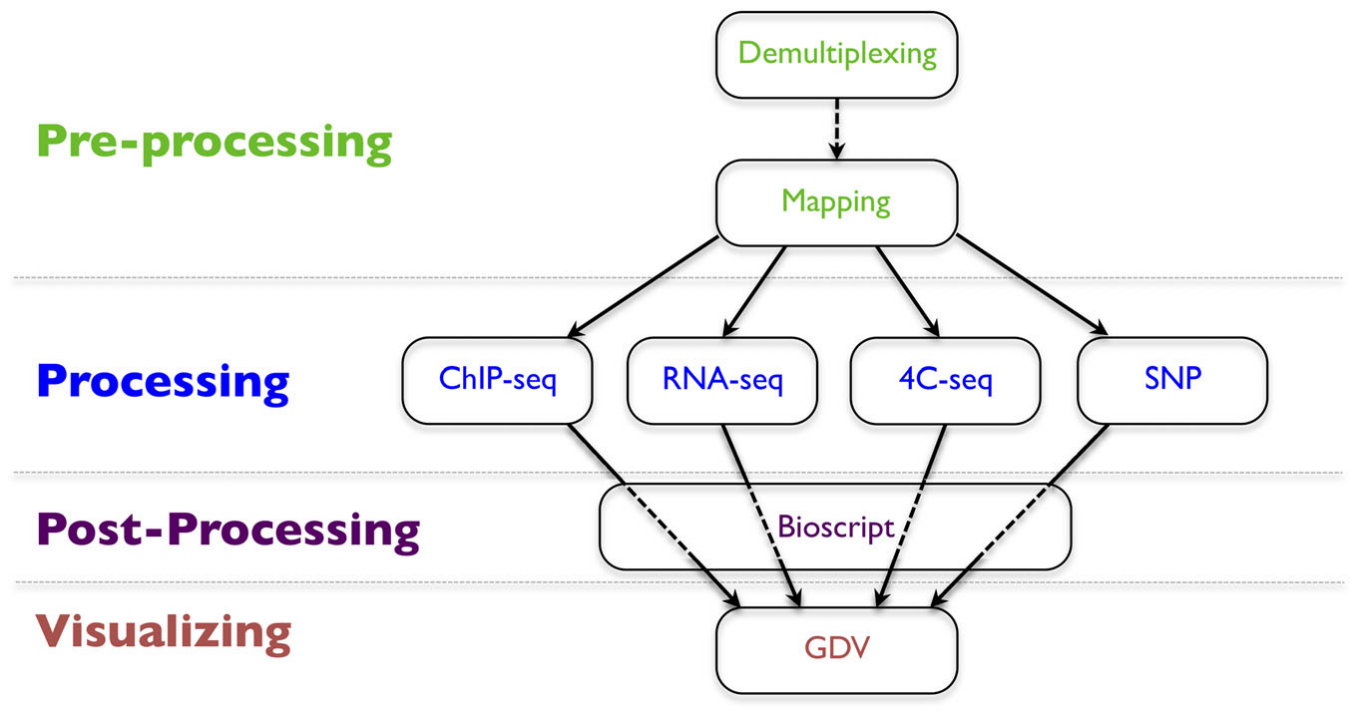
HTSstation modules. Different workflows are obtained as combinations of the two pre-processing modules (dealing with raw sequences) and the four application-specific modules (manipulating sequence alignments and genomic profiles). Results can be visualized using our genomic browser GDV or sent to external post-processing tools for downstream analysis.

HTSstation workflows rely on interconnected modules revolving around the sequence mapping which uses Bowtie [16] (upgraded to Bowtie2 since September 2013) to map sequencing reads to a reference genome or sequence database and calculates genome-wide coverage profiles. This mapping module is preceded by an optional demultiplexing step, and followed by application-specific modules (ChIP-seq, RNA-seq, 4C-seq and SNP calling, see Figure 1). Each module returns a table of output files that can be downloaded, sent to external tools for viewing and further processing.

**Figure 2:**
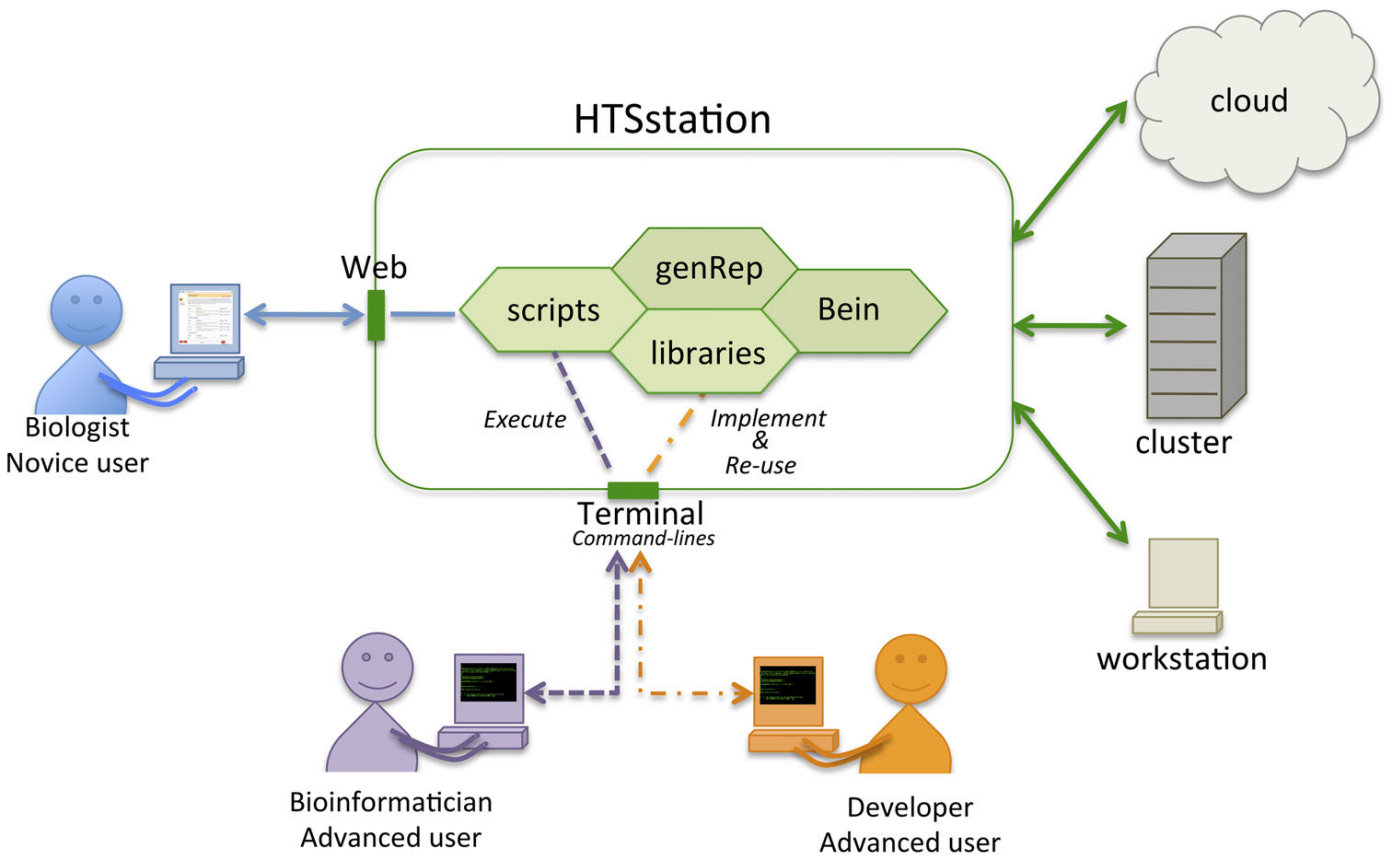
Architecture of HTSstation. This platform offers different access modalities. The web interface targets biologists and occasional users. Command-line execution of underlying executables (bbcfutils) will fit the needs of more advanced users. Users with programming skills can import the libraries (bbcflib) and implement new workflows. GenRep (genomic repository) provides consistency and versioning of reference genome data and Bein (workflow manager) handles dispatching, tracking and documenting programs executions and outputs in a computing environment-independent manner: analyses can be launched locally, or dispatched to a cloud or a cluster.

**Figure 3:**
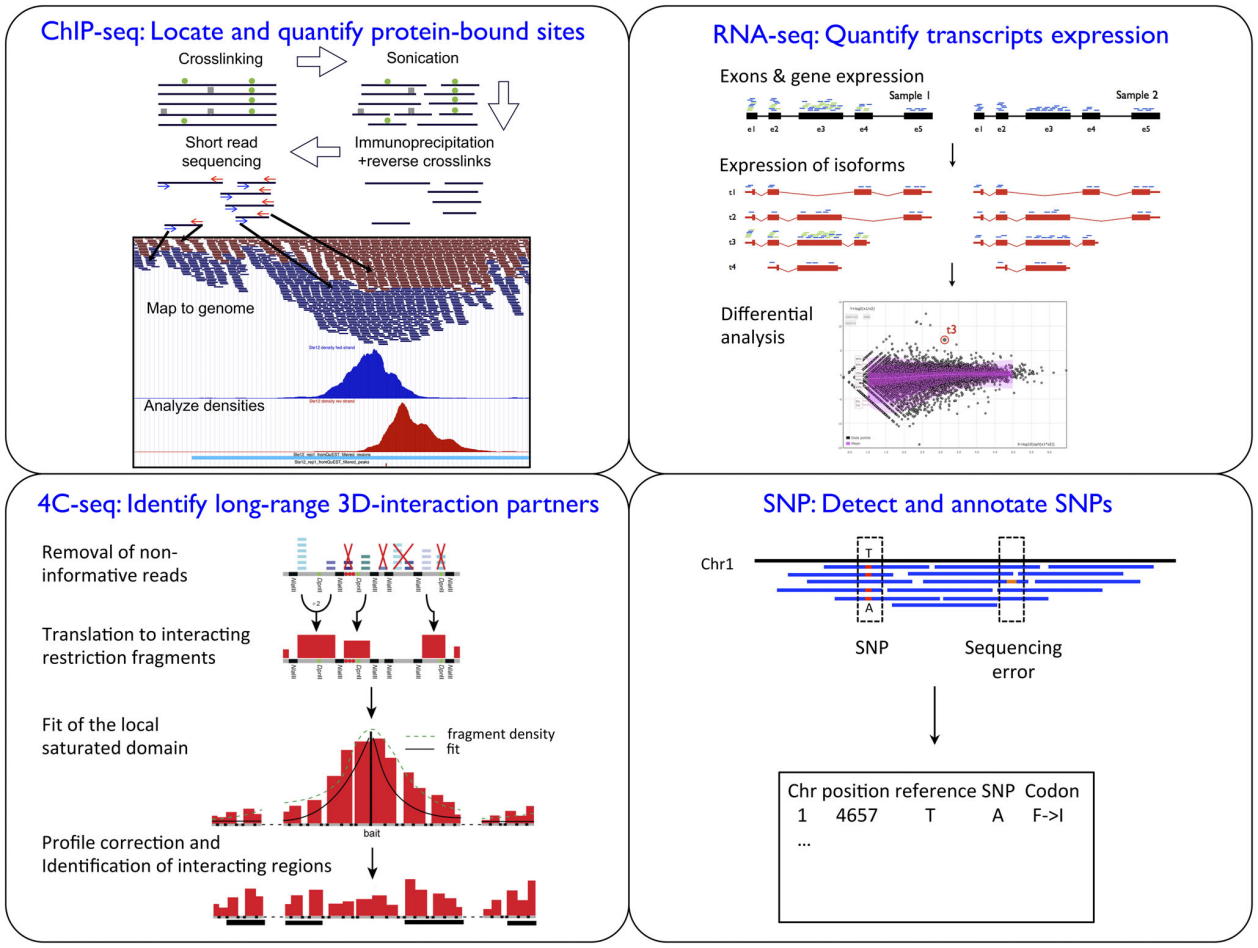
Summary of the application-specific analyses. Each module is dedicated to the analysis of a particular type of HTS experiment. The ChIP-seq module seeks significant interactions of protein with DNA, the RNA-seq module quantifies the expression levels of transcripts and compares them between conditions, the 4C-seq module identifies physical interactions along the DNA sequence and the re-sequencing module discovers polymorphisms.

The overall design of HTSstation strives to accommodate three types of user experiences (see Figure 2). Biologists will mostly interact with the web interface made of intuitive and simple web forms requiring minimal knowledge of the specifics of the underlying programs and algorithms (Figure 3 and File S2, panel A). Bioinformaticians will typically prefer a scripting approach with detailed control over the back-end process. Each module has its own command-line Python program with a number of specific flags and options. Lastly programming enthusiasts will be able to download and extend our source code from our public repository (programs are at https://github.com/bbcf/bbcfutils/ and libraries at https://github.com/bbcf/bbcflib/). Each workflow script consists in a succession of elementary operations defined as library functions. Other workflows can be built by combining some of those operations with new ones and in particular incorporate third-party tools via system calls.

Many functionalities developed for HTSstation are useful in a broader context and have been designed as independent applications: our generic workflow manager, Bein, handles tracking files, logging and dispatching program executions to (in our case) a computing cluster. The GenRep application (http://bbcftools.epfl.ch/genrep) manages genome references and their associated files (Bowtie indexes, FASTA sequence files and GTF annotation files) with a versioning system. We also integrated a customized genome browser (GDV: http://gdv.epfl.ch) and a collection of statistical analysis and data mining algorithms provided under the Bioscript collection (http://bbcftools.epfl.ch/bs); see Methods section and File S1 for more details.

## Results

To illustrate the analysis capabilities of HTSstation, we detail below a number of specific cases that we have selected from relevant publications having used early versions of these pipelines. All corresponding user forms and results are available online (http://htsstation.epfl.ch/examples), together with tutorials and technical documentation.

**Figure 4:**
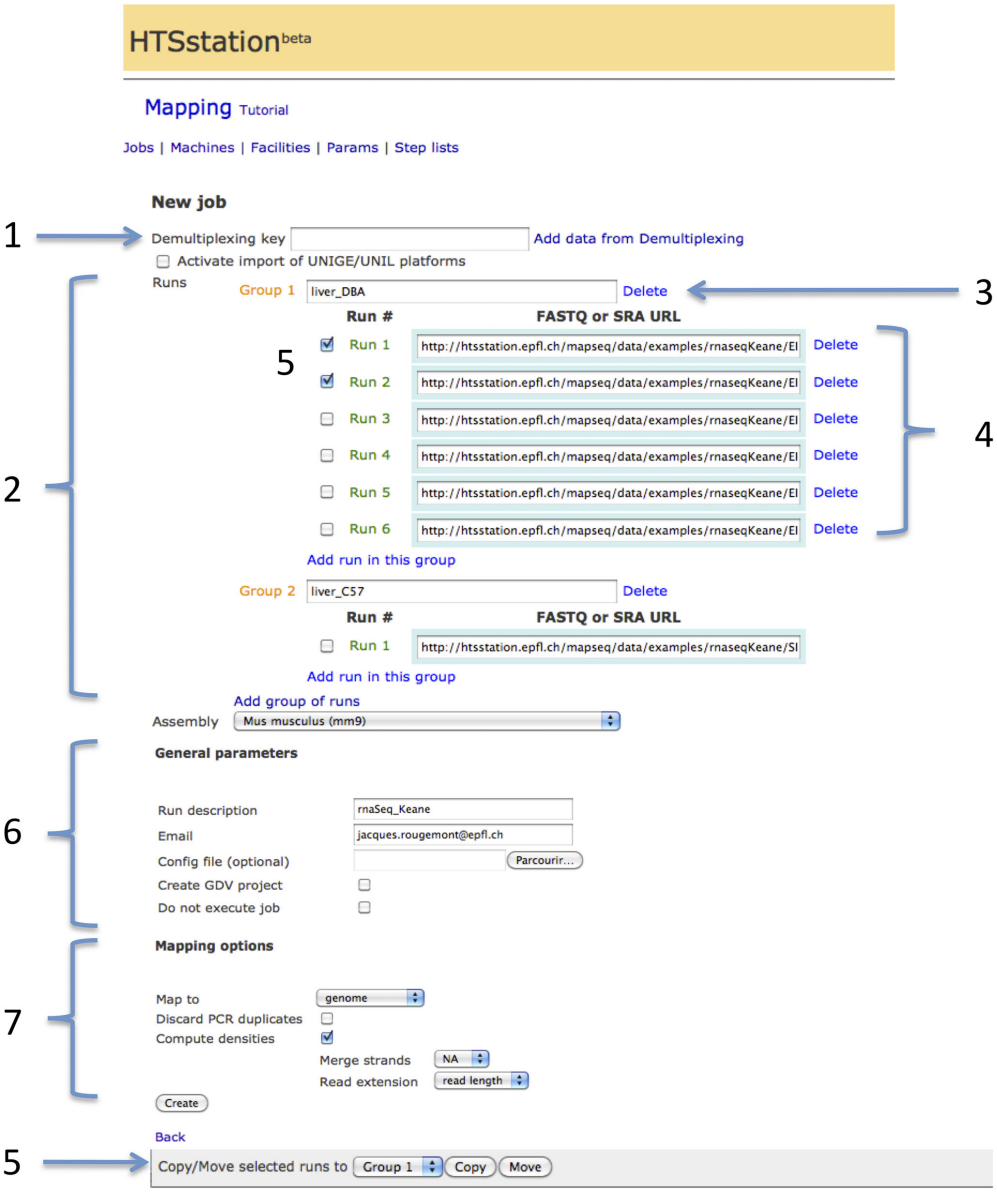
Design of the web interface. Input form of the mapping module: results from a previous job are imported using its unique key (1), e.g. from a demultiplexing task. Users provide inputs as URLs and organize them hierarchically in groups and runs (2) where groups represent distinct experimental conditions (3) and runs are distinct replicates (4). When relevant, some groups can be selected as representing the control condition. Selected runs can be moved between groups (5). All modules have a section with general parameters (e.g. email and analysis name) (6) and module-specific options (7).

All analyses follow the same schema (see Figure 4) by defining one or several experimental conditions each including one or several replicates and a reference genome (picked from a list of standard genomes maintained on the GenRep server or as a user-provided FASTA file). Each analysis is attributed a unique random execution key which is used to access the results page upon job completion, but also to transfer results of one pipeline (*e.g.* mapping) to another (*e.g.* RNA-seq, see File S2, panels B–C).

### Analysis of Kap1 repression of endogenous retroviruses in Mouse ES cells

The transcriptional repressor *Kap1* was shown to mediate silencing of endogenous retro-viruses (ERV) in mouse embryonic stem (ES) cells [7], as deduced from comparing the transcriptomes of wild-type and *Kap1* knockout ES cells. Two replicates of the KO condition and one of the WT were generated producing three FASTQ files containing a total of 47 M Illumina GAII sequencing reads. We submitted these files to HTSstation mapping module to generate three BAM files of mappings to the mm 9 mouse genome and genome-wide coverage densities as BIGWIG files. The default options use a seed of 28 bp with 2 mismatches and a maximum of 5 hits per read. These mappings were then transferred to the RNA-seq module to compute differential isoform expression analysis. This proceeds through every Ensembl [17] annotated exon and computes its average read coverage, then infers expression of every Ensembl transcript from its exon composition (see File S1).

**Figure 5:**
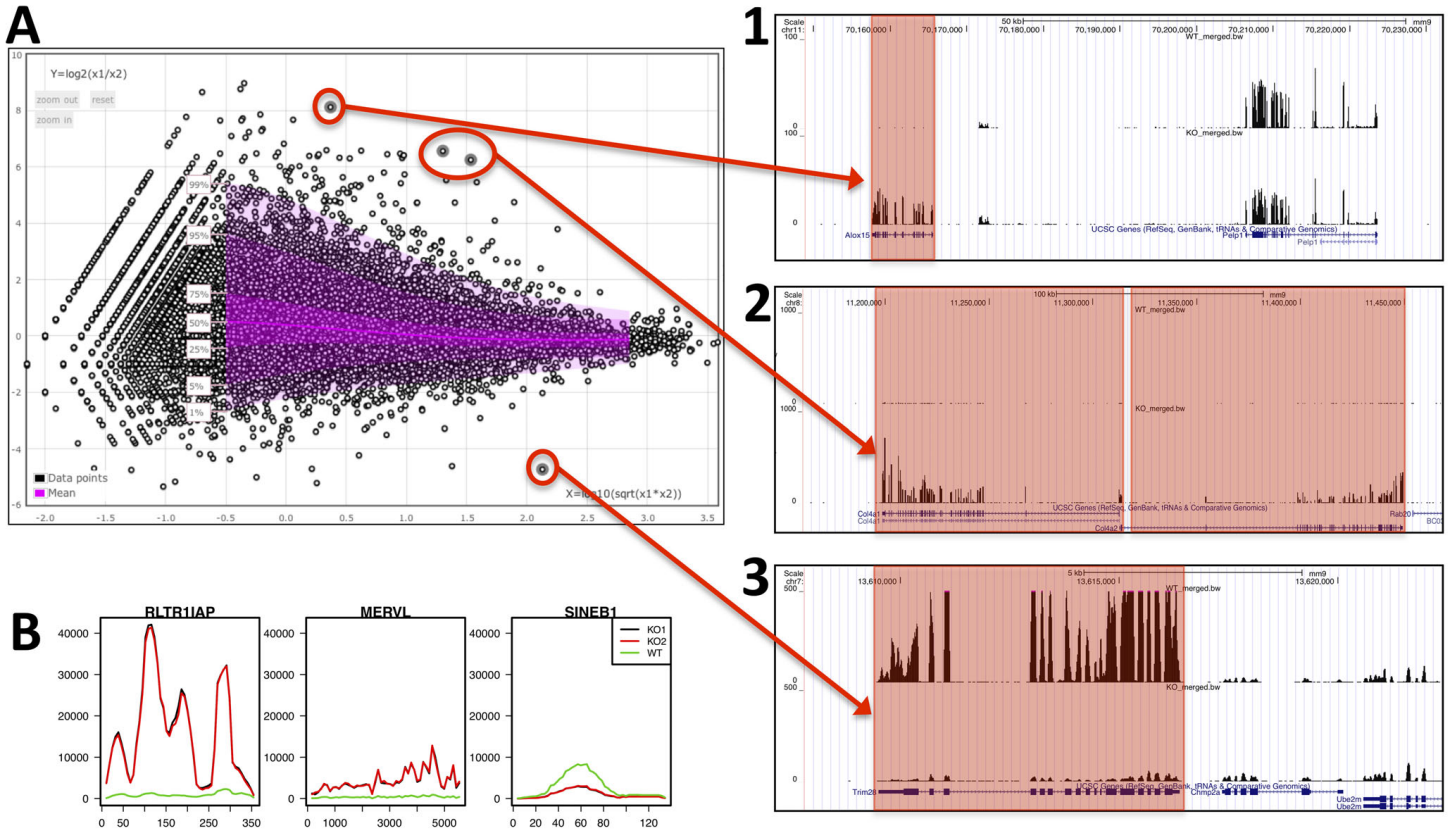
Genomic and differential analysis views of RNA-seq data. **A**) Differential expression analysis of coding genes (MA-plot) shows a few significantly differential genes which is consistent with the signal displayed along the genome: **1**) *Alox15*, on chromosome 11 is strongly over-expressed in the KO condition, unlike the next gene downstream (*Pelp1*) **2**) *Col4a1* and *Col4a2* (located next to each-other on chromosome 8) are both over-expressed in the KO condition, **3**) *Trim28*/*Kap1* is the knocked-out gene and is consistently strongly suppressed. Remark that the exon structure of the genes appears clearly in the genomic view. **B**) Reads were also mapped and quantified on the Repbase collection of repetitive elements. Plots of read coverage along the sequence of three representative examples show that *RLT1IAP* is highly over-expressed in KO mice, *MERVL* is slightly over-expressed and *SINEB1* is under-expressed.

Differential expression analysis between the two conditions was performed using the R package DESeq [18] and the results can be viewed as an interactive MA-plot highlighting interesting genes such as *Alox15, Col4a1* and *Col4a2* which are over-expressed in the KO condition as can be verified by direct inspection in the UCSC genome browser and, obviously, the *Kap1* gene itself stands out as the most down-regulated gene on the MA-plot (Figure 5A).

A complementary analysis was performed with the same reads by mapping them to the Repbase collection of repetitive DNA elements (rodent section, version 16.08 [19]) and analyzed similarly to genes for differential expression, to reveal induction of ERV elements in the KO condition. This illustrates the versatility of the mapping tool which accepts any collection of sequences in input, beyond the standard genomes maintained in GenRep. Moreover, using the post-processing plotting functions we generated coverage profiles along a selection of these elements demonstrating the quantitative difference between the two conditions (Figure 5B).

### The phylogeography of *Mycobacterium leprae*

The phylogeography of the human obligate pathogen *Mycobacterium leprae* was established by genotyping more than 400 samples including 5 complete genomes [3], [8]. We have mapped the Illumina GAII reads for all 5 strains (directly from their SRA urls) against the reference TN strain (refseq NC_002677.1) obtaining an average coverage between 0.3× and 2×.

The resulting BAM files were subsequently analyzed with HTSstation's SNP module, to extract high-confidence single nucleotide polymorphisms (SNPs) using SAMtools [20]. Only high-coverage regions were analyzed and statistical support for each polymorphism is taken into account to filter out false positives. Then each SNP is linked to its genomic context using annotations available for the target genome. A list of 172 snp positions including 64 non-synonymous coding variants was found.

### The dynamic architecture of *Hox* genes clusters

During mouse developments, transcriptionally active and inactive genes of the *Hox* clusters segregate into distinct 3-dimensional domains, and upon activation genes shuttle from one compartment to the other upon activation [1]. To demonstrate that, the authors collected Circular Chromosome Conformation Capture followed by HTS sequencing data (4C-seq), including *Hoxd13* and *Hoxd4* viewpoints in two mouse embryonic tissues: forebrain (FB) where both genes are inactive, and anterior trunk (AT) where *Hoxd4* is active but not *Hoxd13.* The 4C data was complemented with Chromatin Immuno-precipitation and sequencing data (ChIP-seq) that characterizes histone modifications associated with transcriptional activity (H3K4me3) and transcriptional repression (H3K27me3) in the same tissues.

The 4C-seq data were first demultiplexed (using viewpoint-specific primer sequences) then mapped onto the mouse genome (mm9) with Bowtie. These mappings were then imported into the 4C-seq application and compared to a pre-computed library of NlaII restriction fragments [1]. The normalized read counts per restriction fragments were corrected for local interaction profile and a smoothed density profile generated (moving average of 29 fragments per window) to which a domainogram algorithm [1], [21], [22] was applied to identify long-range interacting regions.

**Figure 6:**
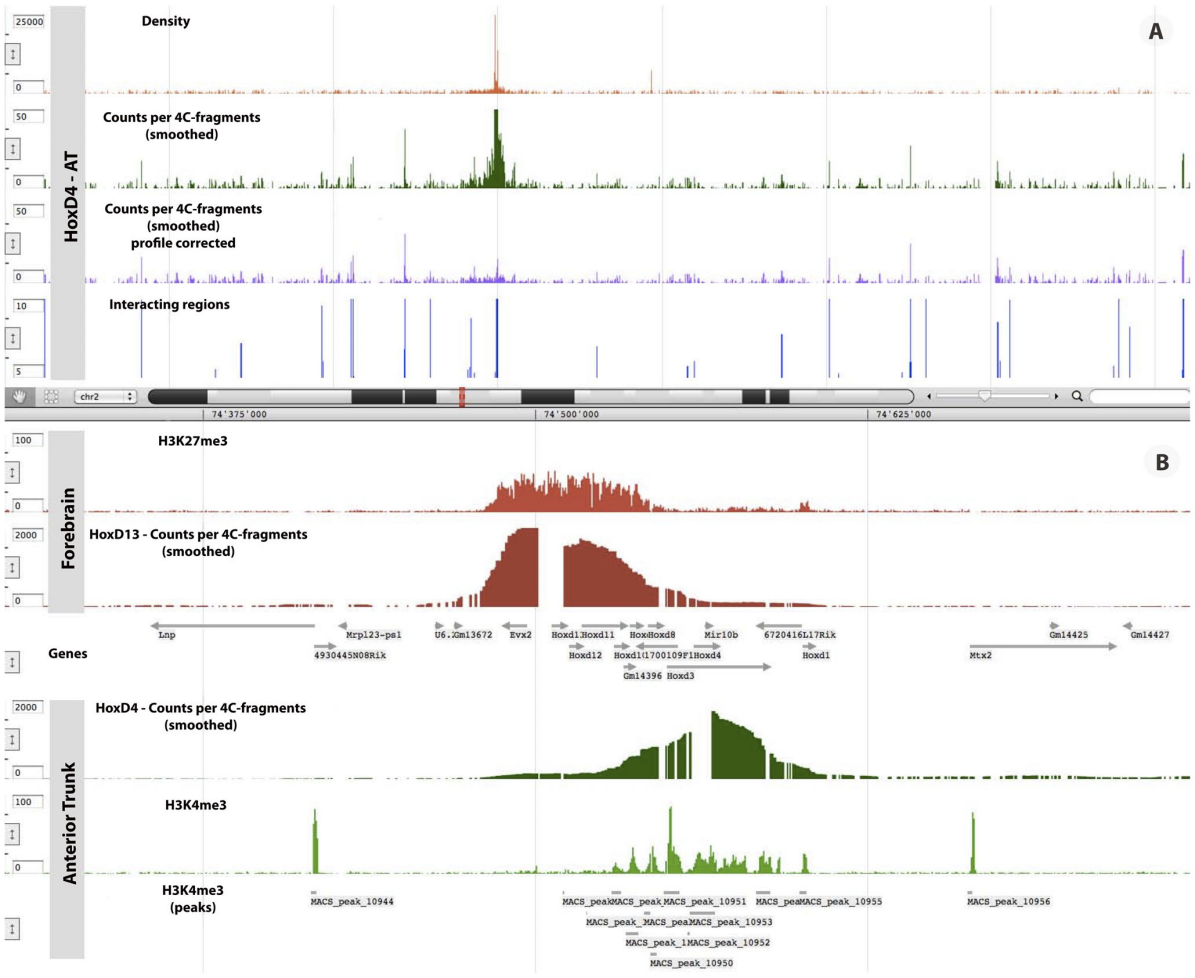
Chromatin configuration and transcriptional status in the *HoxD* cluster. **A**) Illustration of the successive steps of a 4C-seq analysis. From top to bottom: coverage profile (reads density) is smoothed by a moving average. The smoothed density is corrected by fitting a model of the local interaction profile and long-range interacting regions are identified with the domainogram algorithm. **B**) From top to bottom: smoothed 4C-seq profiles for *Hoxd13* and *Hoxd4* in AT; the ChIP-seq density profiles of H3K27me3 and H3K4me3 in the same tissues with the peaks of H3K4me3 found by MACS. This figure was generated with the GDV genome viewer.

Figure 6A illustrates those successive steps for the *Hoxd4* viewpoint. This view emphasizes the localized effect of the profile correction (compare the two middle tracks: raw results after window smoothing in green and profile-corrected data in purple), which does not affect the identification of potential interacting partners in the vicinity of the viewpoint (bottom track).

Read densities for the ChIP-seq data were computed by first extending reads to 100 bp and next merging strand-specific densities shifted downstream (in read orientation) by 75 bp to center relative to the average size of sonication fragments. Unlike previous examples, PCR duplicates were removed after mapping and prior to quantification (specifically only one read per position and strand was retained). Peak detection was performed for the active mark H3K4me3 in each tissue sample by running the MACS software [23].

Figure 6B shows a combined view of the 4C-seq smoothed data for *Hoxd13* and *Hoxd4* viewpoints in AT, together with the density profiles of H3K27me3 and H3K4me3 in the same tissue, as well as the peaks found for H3K4me3. In this figure, both the active and inactive compartments appear clearly, with a boundary situated around gene *Hoxd8*, which is moving from the inactive to the active state in AT.

**Figure 7:**
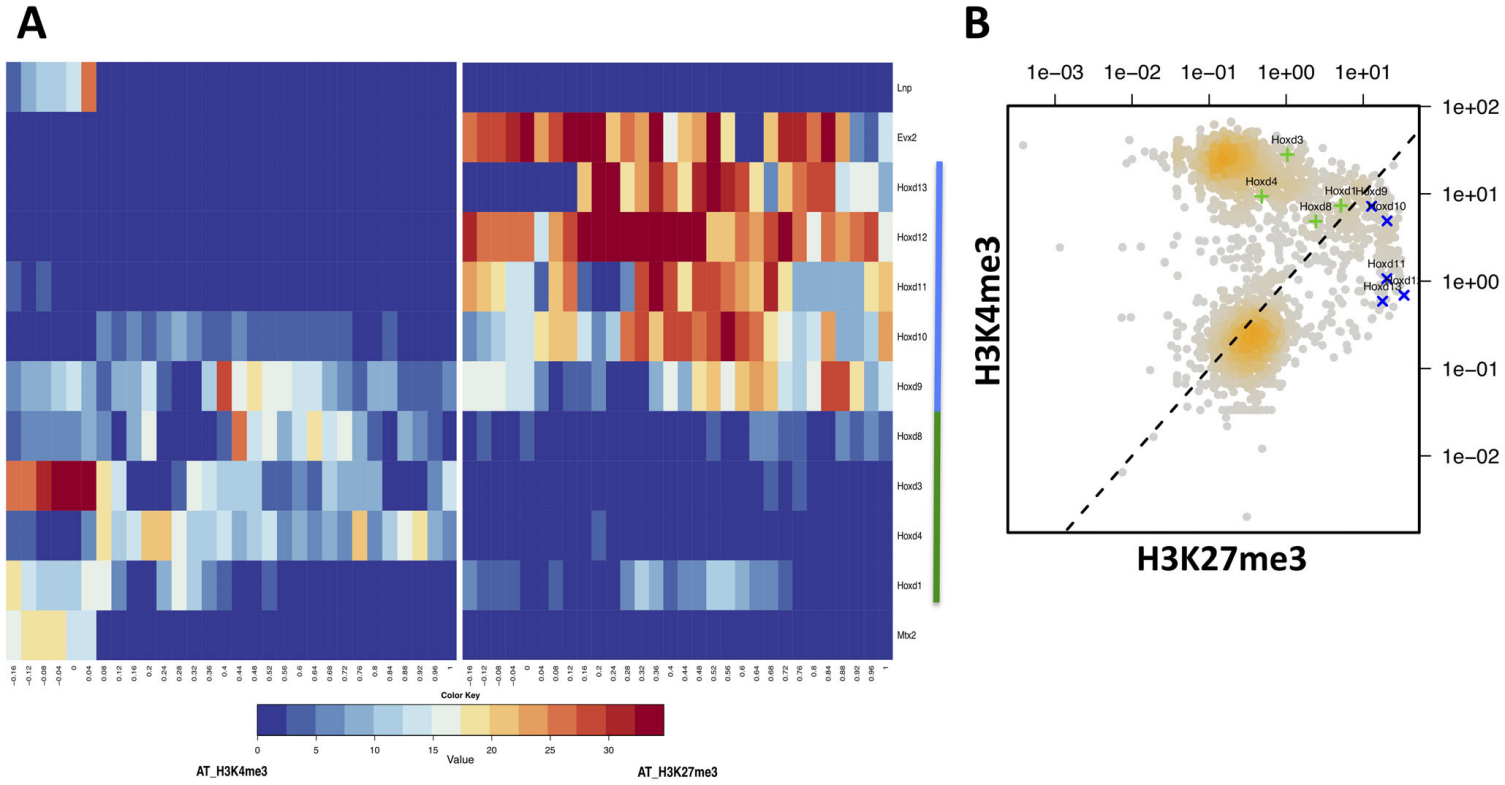
Global analysis of chromatin state. **A**) Heatmaps of *HoxD* genes segmented into 25 bins plus 5 bins of 20 bp in the gene promoters. Left panel contains the H3K4me3 average ChIP-seq values; right panel shows the same for H3K27me3. The blue bar indicates the inactive genes, the green bar the active genes. **B**) Scatter plot showing all genes of chromosome 2 (2 Kb segments around gene starts, the density of genes is indicated by the intensity of orange) with the *HoxD* genes highlighted in green (active segment) and blue (repressed segment).

To illustrate the post-processing capabilities of HTSstation, we selected the H3K4me3 and H3K27me3 AT ChIP-seq data and generated 2 graphical representations: a heatmap of the ChIP-seq signal along genes in and around the *HoxD* cluster shows a clear division between inactive and active sections (left and right of *Hoxd9*, Figure 7A). We then generated a BED file containing 2 Kb intervals around every gene start on chromosome 2 and quantified the ChIP-seq signal accordingly. Plotting the H3K4me3 signal as a function of H3K27me3 on a scatter plot shows a nice division between two roughly equal populations of active and inactive genes (*i.e.,* high and low H3K4me3) and a small number of H3K27me3 positive genes (repressed). The *HoxD* cluster spreads between the repressed/inactive and the active compartments with *Hoxd9* sitting at an intermediary position (Figure 7B).

## Discussion

The HTSstation portal has over its two years lifetime processed in excess of 500 jobs per year with an average of 15 distinct users per month. Complete analyses with complex experimental designs through several HTSstation modules typically take less than two days (ignoring queuing time on the computing cluster). Beyond the tool itself we believe that HTSstation represents an innovative framework for high-throughput data analysis methods that combines high flexibility using ‘off the shelf’ modules. The framework mostly relies on external software packages that are widely accepted standards in the field (Bowtie, SAMtools, DESeq, MACS, SOAPsplice, Exonerate, *etc*.) but takes care of scaling with increasing data volumes, of consistency between inputs and outputs of unrelated tools, and allows us to seamlessly integrate specific workarounds and additions for missing or inadequate features of existing products (see Methods). Moreover the currently selected tools will be easily replaced in the future with their potential successors.

It also integrates in its core design the normal process of bioinformatics development cycle: command-line tools with specifically tuned options are integrated into Bash, Perl or Python scripts that, when stabilized and in need of scaling to a large number of executions, are packaged into modules. Our framework provides a structured method running through each of these steps and eventually provides a web interface for the benefit of the biologist looking for a swift processing of his/her data.

The web interface of HTSstation was deliberately designed for user-friendliness rather than exhaustiveness. We focused on biologically telling options (such as “remove PCR duplicates”) rather than algorithmically inclined options (“length of alignment seed”) for which we have heuristically set predefined values, through the development process described above. However, default program option can be modified by the user: this requires uploading a configuration file (a simple human-readable text file, similar in structure to the INI file format) through the job submission form.

### Accessibility and reproducibility of the results

The choice of a web-based client-server architecture presents an immediate advantage for sharing data: the results of an analysis are presented as web pages with obfuscated URLs. These pages include detailed description of the analysis setup, downloadable file bundles and summary reports. The complete results or any individual file can be shared with collaborators or uploaded to external viewing and processing software via their URLs.

Reproducibility of analyses is facilitated by a cloning functionality which copies parameters and input files from a previous run. Users can then provide new inputs and parameter values. From the system administration point of view, the workflow manager (Bein) stores all execution parameters and links to input and output files in a database. This facilitates the management of pipeline executions also in the context of a direct command line usage of the scripts.

A pipeline builder has been designed to configure a sequence of jobs by filling a single form. Selecting the analysis type determines a list of relevant modules (*e.g.*, demultiplexing, mapping, 4c-seq). It then produces a single form containing all module-specific parameters in a non-redundant fashion. After submission, a daemon executes the successive steps of the pipeline in the background.

### Availability and extensions

Our libraries are mainly developed in Python and this framework is easily deployable locally, in part or completely. The source code is available through the social coding platform Github (https://github.com/bbcf/ – web applications and databases are available upon request). We therefore encourage the involvement and contributions from interested developers at other facilities. HTSstation is thereby destined to evolve, steered by its users towards new applications of High-Throughput Sequencing and new analysis modalities.

## Methods and Implementation

In this section we briefly describe each application available in HTSstation (Fig. 3) and highlight their salient features. Individual tutorials and documentation are provided on the web site and more detailed methods are available in File S1 (see also [24]–[26]).

### Mapping

This module uses Bowtie [16] to map raw sequences to a reference genome and returns the alignments as BAM files [20], as well as the associated genome-wide read densities.

We implemented an optional removal of PCR duplicates at the level of BAM files [24], [26]. This uses a Poisson distribution to estimate the likelihood of obtaining *n* reads at the same position and limits this number to the 95% probability range. Using this option helps reduce the number of false positives in peak detection for ChIP-seq applications. Similarly, we post-process Bowtie's BAM output to include the optional “NH” tag that indicates the number of alignments found for each given read. This is used as a weight when computing genome-wide read densities (using our own Bam2wig application).

A mapping report is generated to provide informative diagnostics such as total number of reads mapped, proportion of alignment by strand, number of mismatches, genome coverage statistics.

### ChIP-seq

The purpose of this module is to analyze ChIP-seq peaks using the MACS software [23]. A novel deconvolution algorithm is provided, which evaluates the shape of peaks within enriched regions found by MACS. This provides a more accurate estimate of binding site location and a lower number of false positives [4]. Users can additionally run motif discovery within these peaks using MEME [27].

### RNA-seq

The RNA-seq procedure uses an original optimization procedure to properly infer transcript expression from quantification at the exon level. The proposed model optimizes the distribution of reads among alternative transcripts, then runs DESeq [18] to infer differential expression of genes and their isoforms between samples. In the results page of the RNA-seq module, an interactive MA-plot can be generated for all pairwise comparisons including replicates of the same condition (see File S2, panels D–E). Discovery of new splice junctions is provided by running SOAPsplice [28] on unaligned reads.

### 4C-seq

The 4C-seq module implements the methods described in [1] and [25] complemented with tools used in the analysis of 4C experiments [29], such as a partitioning algorithm and domainograms [1], [21], [22] to identify regions of enriched interaction frequency. We have also implemented a profile correction algorithm that compensates for non-specific interactions as a function of linear distance from the viewpoint based on the power-law rule described in [30].

### SNPs

This module applies SAMtools pileup [20] to detect discordant bases in reads aligned to a reference genome. We filter out low-coverage regions and infrequent SNPs (with a ploidy-dependent threshold). Finally we annotate the selected SNPs according to their location within genes, and the corresponding amino-acid change for non-synonymous SNPs.

### Post-processing

Bioscript is a Python library of genomic data processing algorithms that can be run from the command line or imported in custom Python scripts. A web service was set up to automatically generate HTML forms for each algorithm. These can be embedded within third party web application, in this case HTSstation, or accessed directly at http://bbcftools.epfl.ch/bs. The client (HTSstation) provides file URI (from the output of, *e.g.* a ChIP-seq or an RNA-seq analysis) to the service (Bioscript) and collects its outputs for download or further processing. On the server side Bioscript deals with the job queues and processing. To name but a few Bioscript currently includes tools to

- Generate descriptive and quantitative statistics and graphics such as heatmaps, correlation plots and MA-plots (see File S2, panel E).
- A set of file manipulation tools including a file format converter (*e.g.*, from WIG to BIGWIG) and a BAM to density generator to create normalized coverage profiles from read alignments.
- A number of analysis tools to annotate (*i.e.*, associate features to genome annotations), cluster, transform (*e.g.*, data smoothing or log transform) or manipulate (*e.g.*, intersect) genomic datasets. This also includes the BEDtools [31] suite, the R packages TopGo [32] (Gene Onotlogy), DEseq (differential expression statistics) and DNA motif scanning on selected genomic regions (typically ChIP-seq peaks).

### Backend

We have designed an SQLite format to accommodate the various file formats used in genomics [15], with transparent import and export functionalities to and from these files (BED, GFF/GTF, WIG, BIGWIG, BAM). Together with the two backbone applications (GenRep and Bein), they not only ensure the reliability and reproducibility of the analysis, but also improve the long-term maintenance and extension to new modules within the platform. In addition, our workflow manager (Bein) completely decouples program executions from their environment (cluster, local workstation), making it easy to adapt the entire system to a new run-time environment, such as a cloud.

### Data Access

All data used in this paper have been published elsewhere [1], [7], [8]. Corresponding data accessions are GEO GSE31570, GSE41903, and SRA SRP001064.

## Supporting Information

File S1 **Detailed methods.**
(PDF)

File S2 **Pictorial description of a complete RNA-seq analysis process.** A. Job description page for the mapping module. The first block defines the data sources and is structured according to experimental design, the second block is a general description usually common to successive jobs in the same analysis sequence, the third block sets module-specific options. B. Mapping results page, with links to the source files, to the results files, and to viewing tools. Notice the unique identifier at the top of the page. C. Processing further to the RNA-seq differential expression analysis involves importing the mapping results into the RNA-seq module using one or more job identifiers. D. RNA-seq results are presented in a similar way to panel B with additional viewing options (MA-plots). E. The interactive MA-plot provides a quick overview of differential expression between two conditions.
(PDF)
