# Supplementary material for: HTSstation: A Web Application and Open-Access Libraries for High-Throughput Sequencing Data Analysis
Source: PLoS One. 2014 Jan 27;9(1):e85879. doi: 10.1371/journal.pone.0085879 (PMC3903476; doi:10.1371/journal.pone.0085879)
Supplement: File S1 — Detailed methods. (PDF) [file pone.0085879.s001.pdf]

HTSstation
Detailed Methods

EPFL - Bioinformatics and Biostatistics Core Facility

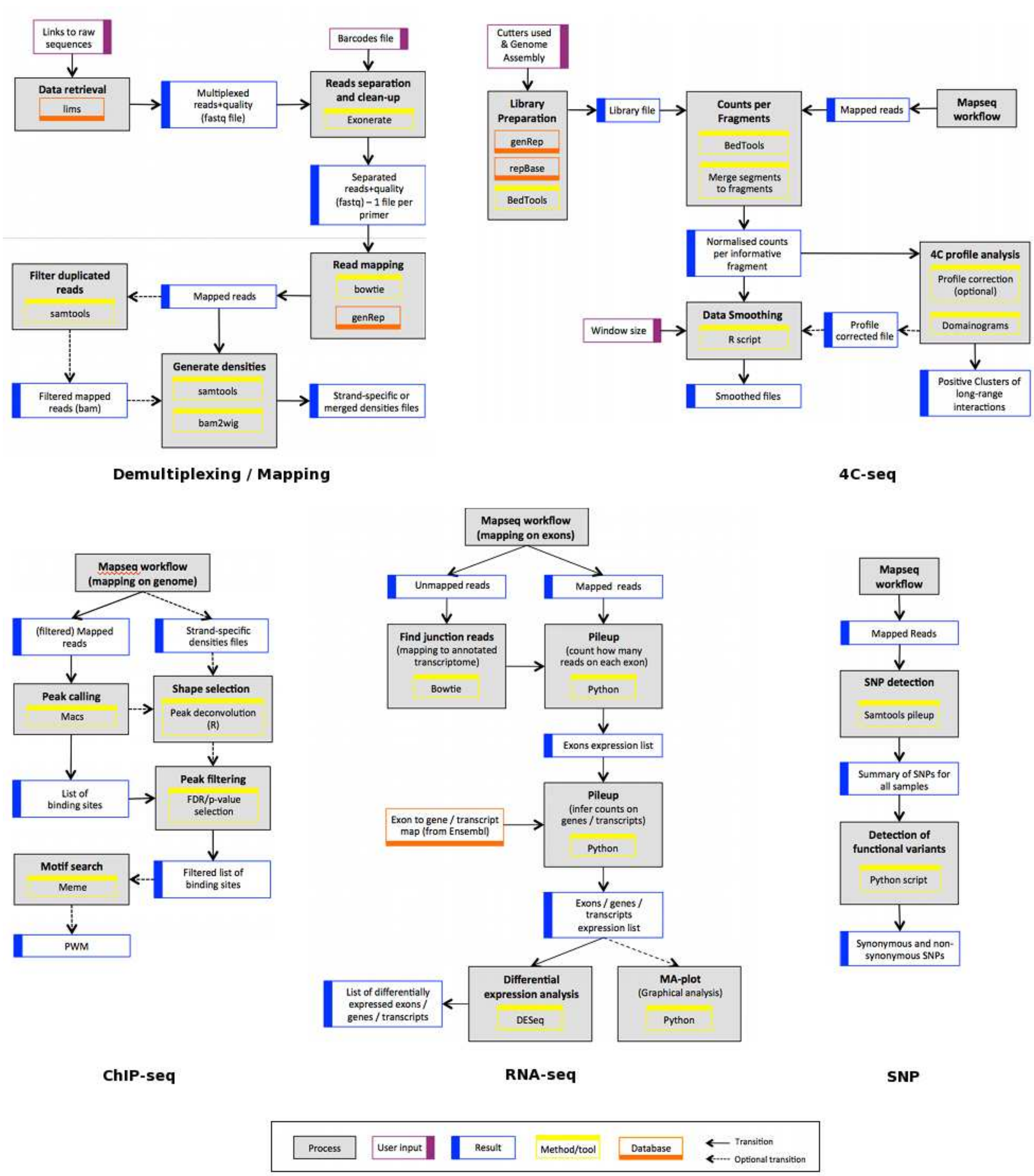

Figure 1: Summary of workflows used in HTSstation

1 GenRep: a genome data repository

The problem of maintaining a consistent set of genome sequences and annotations, and managing updates is as old as bioinformatics itself. While major institutions like NCBI and EBI are making this effort and remain primary sources of data, we needed a system to mirror a relevant selection of genomic data from these resources and relate them consistently. Our goal was to generate and maintain a consistent file hierarchy on our computing servers as required to run our programs. This hierarchy includes:

- Whole genome and exome fasta files,
- Bowtie indexes,
- Genome features (GTF files from Ensembl, converted to SQLite databases),
- Pre-computed genome statistics (nucleotide frequencies, chromosome sizes, etc.)
- Additional genomic data associated with genome sequences (repeat-masking data, etc.),
- Additional functional data associated with species (PWMs for transcription factor motifs).

A web-based interface (Ruby on Rails) is used to manage the mirroring of selected species and assemblies, and to generate the file hierarchy (`http://bbcftools.epfl.ch/genrep/`). A Python library and a REST API are provided to access information from the database and to extract complete paths for each file (e.g. to construct a Bowtie command), or to make specific queries (find genes, features or sequences at specific positions in the genome, retrieve genome statistics, convert between chromosome identifiers and names, etc.).

2 Bein: a workflow execution environment

2.1 Motivation

When a data analysis exceeds the capacity of a spreadsheet, bioinformaticians typically turn to the Unix shell. They proceed by trying commands interactively, and copying those that succeed into a script. Eventually each logical step becomes an independent script which reads data from a file, performs some analysis, and writes its results to another file. The whole analysis proceeds by running script A on file X to produce file Y, script B on file Y to produce file Z, etc.

This way of working is comfortable and flexible, but has several serious drawbacks. For example, to perform identical analyses on several sets of data, the scientist runs his scripts in order on each data set to produce a set of output files. With one or two data sets, it is simple to keep track of which file came from which data set; with more data sets, the process becomes increasingly cumbersome.

Furthermore, the same operation often appears in several analyses, but the script implementing it in one analysis can rarely be used unchanged in another. Modifications are typically needed in the script's input and output rather than in the actual computations it performs, yet the code for the operation cannot easily be shared and re-used.

We identified the need for a tool to handle the many pipelines implemented (and upcoming) in HTSstation, that would be more structured than the shell but less constrained than large pipeline management frameworks (Giardine *et al.*, 2005, Blankenberg *et al.*, 2007, Rowe *et al.*, 2003), sized for pipelines of small complexity and small number of iterations. This is how we implemented Bein, which provides an environment for data analysis more efficient than the Unix shell, but similar in spirit.

Bein's core logic is less than 500 lines long. It is well documented, and we have endeavored to make it easy to learn and install.

2.2 Implementation

Bein is implemented in Python which permits the same interactive exploration as the Unix shell, but improves code readability and maintenance. Logical units of analysis become functions instead of separate scripts, so that it is not necessary to create code to read data and write results.

Python functions are much easier to write and work with than Bash scripts, but bioinformatics relies on preexisting scripts and programs outside Python. Bein provides a framework to wrap them as Python functions with a minimum of code. Using an external program requires four things: a known environment in which to run it, appropriate arguments to run it with, a way to interpret its results, and a way to clean up afterwards. Bein makes the first and last steps unnecessary by isolating each analysis in a unique environment (see Figure 2), so we only need to provide a list of arguments, and a function that extracts and returns a value from the program's output.

Data analysis in Bein is organized into "executions," which are sequences of operations applied to a single data set. Bein runs each execution in a clean, predictable environment by creating an empty directory in which all the operations of the execution are performed, and deleting the directory upon exiting the execution (see Figure 2).

Unless stored somewhere, the products of the execution are then erased. Rather than copy the result files to another directory, because running the same analysis a second time would create a jumble of files as happens with the Unix shell, Bein provides simple repositories, called MiniLIMS, which track files and executions. Each execution is associated with a MiniLIMS. When the execution finishes, it writes all errors and outputs of external programs it ran, and any results that should be preserved to its associated MiniLIMS, which assigns the execution a unique, integer identifier.

All together, these three components – external program bindings, executions, and MiniLIMS repositories – make the code of an analysis compact and easy to read. For example the code in the center of Figure 2 connects to a MiniLIMS named `data`, fetches a fasta file with identifier

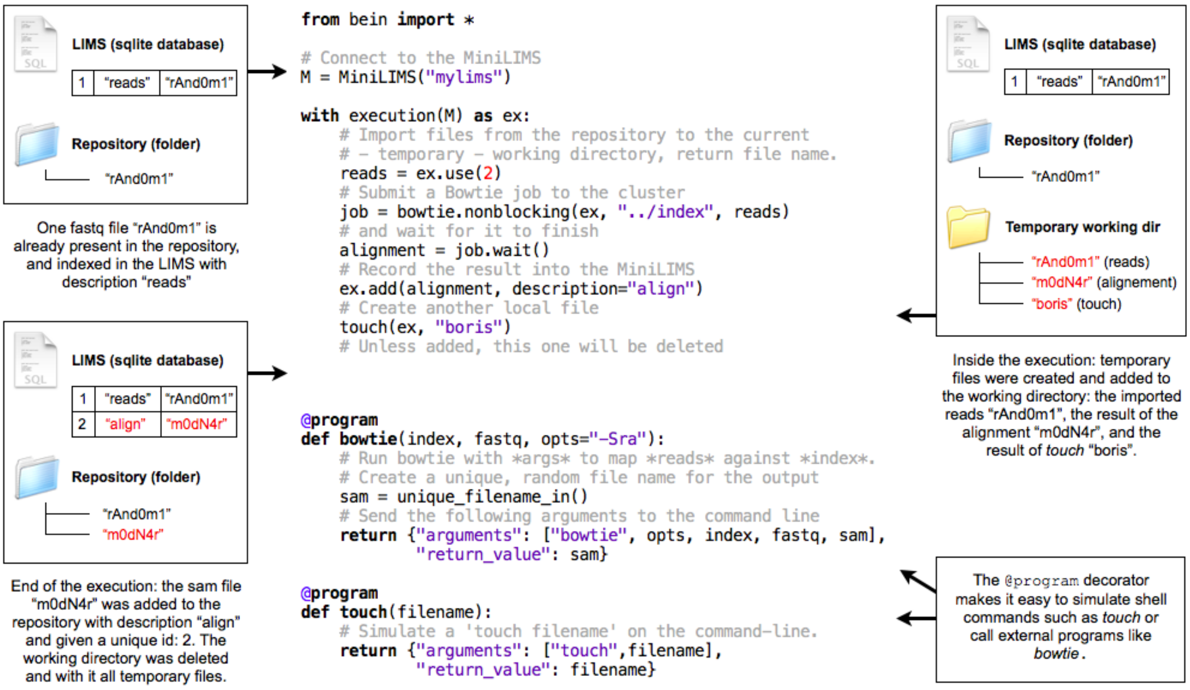

Figure 2: An execution handled by Bein

12 (files are also assigned unique, integer identifiers when added to a MiniLIMS), runs Blast on this file and adds Blast's output to the MiniLIMS. A precious additional feature of Bein is the ability to run external programs in parallel, either locally or by dispatching across a cluster (via submission to the LSF queuing system).

3 Demultiplexing

Our demultiplexing module (see Figure 1) separates multiplexed samples based on the presence of barcodes in the reads and applies a cleaning step to filter reads based on the presence of some specific sequences defined in a primers file. Most sequencing instruments now include their own demultiplexing procedure, therefore this module is mostly used for multiplex 4C-seq experiments.

The separation of samples involves identifying and removing the different bait-specific forward primer sequences at the 5'-end of the sequences. It is based on a global alignment algorithm (Exonerate (Slater and Birney, 2005)), called with the following options: *–model affine:local -o -4 -e -12 -s minScore*. This generates a local alignment with affine gaps, similar to the Smith-Waterman-Gotoh algorithm. The parameters are set with a sufficient stringency to balance between correct classification and falsely discarding reads (see (Slater and Birney, 2005) for more details). By default, they were tuned for a 22nt alignment with up to 2 non-consecutive mismatches or gaps (*-s 77*). In addition, and in order to maximize the accuracy of the results, alignments can be restricted to the first bases of each read (options *-x* and *-n*, defined in the parameter file).

This process returns reads stripped of the barcode and optionally cut at the 3' (low-quality) end, and sorted in one file per primer. Finally, and when appropriate, those reads are also filtered for any sequences specified in the primer file by using Bowtie (Langmead *et al.*, 2009).

4 Mapping

The mapping module (see Figure 1) is using Bowtie (Langmead *et al.*, 2009) with the following options *-Sam 20 –best –strata –chunkmbs 512* and output is converted to BAM format (Li *et al.*, 2009).

During the process of conversion, we optionally remove duplicated reads according to the following rule: at most n reads per strand-specific genomic position will be kept, where n is computed as the 95% percentile of a Poisson distribution with mean equal to the expected genome coverage multiplied by an enrichment factor of 50.

We also add an *NH* flag to each BAM record to indicate the number of alternative alignments found for the same read (*e.g.* if read R has 5 different positions on the genome, the BAM record will contain the flag *NH:i:5*).

Then read alignments are converted to genome-wide densities using a custom C program Bam2wig. This will provide genomic position-specific reads counts normalized by total number of reads (in units of 10 millions), where each of the *NH* alternative mappings is counted as $1/NH$. Reads are extended to a user-specified length (by default the read length of the sequencing run) and either two strand-specific densities are generated, or a single merged density with the option of shifting each strand-specific density by S bases downstream before taking their average. In ChIP-seq applications, S should be chosen equal to half the difference between average sequencing fragment length et read extension.

5 ChIP-seq: peak deconvolution algorithm

The complete ChIP-seq workflow is depicted in Figure 1. We detail below the specific deconvolution algorithm which is used to evaluate the shape of peaks and eliminate false positives as well as increase the spatial resolution of the peaks.

High-throughput sequencing generates $5'$-tags from libraries of double-stranded DNA fragments. In a ChIP-seq experiment, these $\approx 200-400$ bp fragments are produced by sonicating cross-linked genomic DNA, then immuno-precipitating with an antibody specific to a protein of interest before reversing the cross-links. The $5'$-tags are then mapped back to the genome producing two separate counts distributions $\mathrm{P}^{+}_{\mathrm{tag}}(k)$ (on the forward strand) and $\mathrm{P}^{-}_{\mathrm{tag}}(k)$ (on the reverse strand). The $\mathrm{P}^{+}_{\mathrm{tag}}$ distribution is expected to display peaks upstream of the actual binding sites, generated by the $5'$ ends of all fragments overlapping a binding site, and similarly the peaks of $\mathrm{P}^{-}_{\mathrm{tag}}$ will be located on the other side of the binding sites (upstream on the reverse strand).

5.1 Fragment length distribution

We assume that every position in a chromosome of length L is equally likely to break and that the chromosome is fragmented into Lq pieces (of average length $1/q$). If all breaking events are independent, then the length distribution is geometric with parameter q:

$$\mathrm{P}^{(0)}_{\mathrm{len}}(\lambda) = q(1-q)^{\lambda-1} .$$

To reduce the number of parameters and avoid the complication of boundary effects, we set $L=\infty$.

The experimental protocols typically include selecting only fragments with length in a specified range $[\mu,\nu]$. In the remainder of the discussion, we set $\nu=\infty$ since this is an exponentially small perturbation. However, only fragments of size $\lambda \geq \mu$ can be observed. The effective length distribution is therefore

$$\mathrm{P}_{\mathrm{len}}(\lambda) = \begin{cases} 0 & \text{if } \lambda < \mu \ , \\ q(1-q)^{\lambda-\mu} & \text{if } \lambda \geq \mu \ . \end{cases}$$

5.2 Strand-specific reads distribution

We write the two count distributions as convolutions with a fixed kernel $\mathcal{M}$ (see Figure 3):

$$\mathrm{P}^{\pm}_{\mathrm{tag}}(n) = \sum_{k>n-\ell} \mathcal{M}\big(\pm(k-n)\big)\mathrm{P}_{\mathrm{bind}}(k) \ . \tag{5.1}$$

The fraction of transcription factors bound at position k in the genome is $\mathrm{P}_{\mathrm{bind}}(k)$ and we compute the probability $\mathrm{P}^{-}_{\mathrm{base}}(b)$ that a sonication fragment overlapping k breaks at $b \geq k$, thereby producing a count at position b on the negative strand. Such a fragment can start at any position $a \leq k$ having length $\lambda = b-a+1$. The probability of such an fragment is therefore:

$$\begin{aligned} \mathrm{P}^{-}_{\mathrm{base}}(b) &= C^{-1}\sum_{k\leq b}\sum_{a\leq k}\mathrm{P}_{\mathrm{len}}(b-a+1)\mathrm{P}_{\mathrm{bind}}(k) \\ &= \sum_{k\leq b}\mathcal{K}(b-k)\mathrm{P}_{\mathrm{bind}}(k) \ , \end{aligned}$$

where

$$\mathcal{K}(n) = C^{-1}\begin{cases} 1 & \text{if } 0 \leq n < \mu \ , \\ (1-q)^{n-\mu} & \text{if } n \geq \mu \ , \end{cases}$$

and C is a normalization constant to ensure $\sum_n \mathcal{K}(n) = 1$.

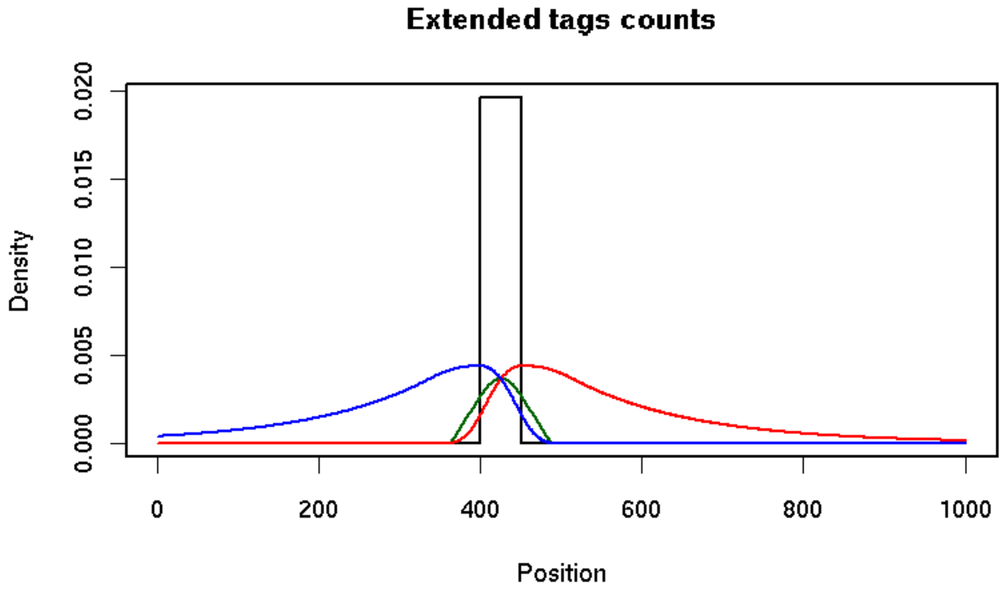

Figure 3: The distributions $\mathrm{P}^+_{\mathrm{tag}}$ (blue) and $\mathrm{P}^-_{\mathrm{tag}}$ (red) generated by a constant binding distribution $P(k)$ (black) in $[400, 450]$ with parameters $\mu = 80$, $q = 1/150$, and $\ell = 36$. The green curve is the geometric average of the two strand-specific distributions.

If we now take into account that tags have a length $\ell < \mu/2$ (typically $\ell = 50$bp) and therefore cover ℓ positions downstream of the break point, the distribution is affected as follows:

$$\begin{aligned} \mathrm{P}^+_{\mathrm{tag}}(n) &= C^{-1} \sum_{a=n-\ell+1}^{n} \mathrm{P}^+_{\mathrm{base}}(a) \\ &= \sum_{k>n-\ell} \mathcal{M}(k-n)\mathrm{P}_{\mathrm{bind}}(k) \ , \end{aligned} \tag{5.2}$$

with

$$\mathcal{M}(n) = C^{-1} \begin{cases} (\ell+n)q & \text{if } 1-\ell \le n < 0 \ , \\ \ell q & \text{if } 0 \le n \le \mu-\ell \ , \\ q(\mu-n)+1-(1-q)^{n+\ell-\mu} & \text{if } \mu-\ell < n < \mu \ , \\ (1-q)^{n-\mu}(1-(1-q)^{\ell}) & \text{if } n \ge \mu \ . \end{cases}$$

We next propose an algorithm for de-convoluting Eq. 5.2 for both strands simultaneously, meaning we solve the following matrix equation for $\mathrm{P}_{\mathrm{bind}}$

$$\begin{pmatrix} \mathrm{P}^+_{\mathrm{tag}} \\ \mathrm{P}^-_{\mathrm{tag}} \end{pmatrix} = \begin{pmatrix} \mathcal{M}^T \\ \mathcal{M} \end{pmatrix} \cdot \mathrm{P}_{\mathrm{bind}} \ ,$$

with the constraints

$$\sum_k \mathrm{P}_{\mathrm{bind}}(k) = 1 \ , \ \text{and } \mathrm{P}_{\mathrm{bind}}(k) \ge 0 \ \forall k \ .$$

Here we represented the convolution kernel as a matrix $\mathcal{M}_{ij} = \mathcal{M}(i-j)$. We solve this problem by searching for the vector $\mathrm{P}_{\mathrm{bind}}$ which minimizes the error

$$\mathcal{E}_0^2(\mathrm{P}_{\mathrm{bind}}; \mu, q, \ell) = \left\| \mathrm{P}^+_{\mathrm{tag}} - \mathcal{M}^T \mathrm{P}_{\mathrm{bind}} \right\|^2 + \left\| \mathrm{P}^-_{\mathrm{tag}} - \mathcal{M}\mathrm{P}_{\mathrm{bind}} \right\|^2 \ .$$

We use quadratic programming (Goldfarb and Idnani, 1983) to find such a minimizer satisfying the constraints. This is applied within each enriched region detected *a priori* by MACS (Zhang *et al.*, 2008). The error $\mathcal{E}_0^2$ provides a confidence estimate for the peak and the spread of the distribution $\mathrm{P_{bind}}$ gives an improved estimate of the binding location.

6 RNA-seq: transcript-level expression estimation

RNA-seq reads are generated from transcribed sequences, and there are several possible options for how to relate them to a known genome sequence (Pepke *et al.*, 2009). We assume a pre-existing annotation of the genome: a set of exon coordinates on the genome, a set of transcripts joining one or several exons, and a set of genes generating one or several transcripts. The set of exon sequences is called the exome (a subset of the genome), and the set of full transcript sequences is called the transcriptome (not a subset of the genome).

Our RNA-seq module takes as input BAM files (see Figure 1) containing the alignment of reads on the genome (or the exome). It counts the number of reads mapped to each exon, and calculates the RPKM score (Reads per Kilobase per Million) ratios defined as follows:

$$\text{RPKM} = \frac{\text{number of reads on the exon}}{10^{-3}\text{exon length} \times 10^{-6}\text{number of reads in the experiment}} .$$

The RPKM of a gene is a straightforward weighted average of the RPKM of its exons. The RPKM of a transcript is harder to calculate. Since an exon can be used to build several different transcripts, it is not clear to which transcript one should attribute a read mapping on that exon. A first possibility consists in attributing a count of $1/k$ to each of the k alternative transcripts. This way one can correctly compute the RPKM of a gene by summing the scores of all its transcripts, but the scores of the transcripts themselves are likely to be wrong. We instead use a mathematical approach of inferring transcripts expression from exons RPKM, by optimizing the distribution of the reads among transcripts.

At the end of the pipeline, scores are normalized between samples using the method of DESeq's "size factors" (Anders and Huber, 2010). We then look for differential expression of genomes between samples using DESeq (on raw read counts).

6.1 Gene expression

Gene G contains n exons $E_1,\dots,E_n$, and let $e_1,\dots,e_n$ be the respective exons RPKM. Let ℓ_k be the length of exon k, and L the length of G. Then the RPKM g of G is given by

$$g = \frac{1}{L}\sum_{k=1}^{n} e_k \cdot \ell_k$$

6.2 Isoform expression

Gene G contains n exons $E_1,\dots,E_n$, and can generate up to $m \leq \sum_{k=1}^{n}\binom{n}{k} = 2^n - 1$ alternative transcripts $T_1,\dots,T_m$. Call $e_1,\dots,e_n$ the (measured) exons RPKM, and $t_1,\dots,t_m$ the (unknown) transcripts RPKM.

For each transcript T_i, define t_i as its RPKM, the indicator function $I_{ik} = 1$ if transcript T_i contains exon E_k, 0 otherwise, and e_k as the RPKM of exon E_k. Obviously, $e_k = \sum_i I_{ik}t_i$; in matrix form:

$$\begin{pmatrix} e_1 \\ \vdots \\ e_n \end{pmatrix} = \begin{pmatrix} I_{11} & \cdots & I_{m1} \\ \vdots & & \vdots \\ I_{1n} & \cdots & I_{mn} \end{pmatrix} \begin{pmatrix} t_1 \\ \vdots \\ t_m \end{pmatrix} .$$

Next define L_i the length of T_i and ℓ_k the length of E_k. We may add the constraint that the total number of reads in a gene is the same if counted from exons or from transcripts, meaning $\sum_k e_k \cdot \ell_k = \sum_i t_i \cdot L_i$.

The linear system then becomes

$$E = \begin{pmatrix} e_1 \\ \vdots \\ e_n \\ \sum_{j=1}^{n} e_j \cdot \ell_j \end{pmatrix} = \begin{pmatrix} I_{11} & I_{21} & \cdots & I_{m1} \\ \vdots & & & \vdots \\ I_{1n} & I_{2n} & \cdots & I_{mn} \\ L_1 & L_2 & \cdots & L_m \end{pmatrix} \cdot \begin{pmatrix} t_1 \\ \vdots \\ t_m \end{pmatrix} = M \cdot T \ .$$

The matrix M will not be invertible and n is typically greater than m (overdetermined system), so we look for a least-square minimizer

$$\min_T ||E - MT||_2^2 \ .$$

Moreover, we search a non-negative solution T using a Python equivalent of Matlab's `lsqnonneg` function[1], itself inspired by an algorithm of Lawson and Hanson (Lawson and Hanson, 1974), p.161. This algorithm finds a positive local minimizer T in about $m/2$ iterations for a typical dataset.

6.2.1 Normalization

One usually wants to compare samples from different experimental conditions $g_1, \ldots, g_l$ each containing several replicates. A typical output of the RNA-seq module is a matrix D of the form

	**g1.1**	**g1.2**	**g1.3**	**g2.1**	**g2.2**	**g2.3**	**g3.1**	**g3.2**	**g3.3**
**feature 1**	178	138	83	1935	53	120	263	928	492
**feature 2**	159	142	112	504	442	528	577	156	1244
**feature 3**	59	19	166	58	1130	826	103	463	852
...	...	...	...	...	...	...	...	...	...

Each numeric column is a different sample, labeled by condition and replicate number. Each line represents a different feature (gene, exon, or transcript).

We use a Python implementation of DESeq's "size factors" (Anders and Huber, 2010) to normalize our samples. The idea is to compare each of our samples to an artificial reference. The transformation applied is the following:

- Compute the geometric mean of each line of D, which gives an $n-$vector.
- Divide each column of D by this mean vector, and store the result in a new matrix, say M.
- Calculate the median of each column of M, which gives an $m-$vector. Its components are called "size factors", and represent the average variation of each sample from their mean.
- Divide each line of D by the vector of size factors.

6.2.2 Splice junctions

Reads spanning splice junction are "lost" during the mapping on exons. Using data from (Rowe *et al.*, 2010), we had 26% of unaligned reads on the genome, 45% of which mapped to the transcriptome, meaning that there were about 12% of reads from splicing sites. Moreover, about half of the remaining (6% of total reads) mapped somewhere else in the genome. Bowtie can save reads that did not map to the genome into a separate fastq file. We map then on the transcriptome attributing each read spanning a splice junction a score of 1/2 to exons on each side of the junction.

[1] `http://diffusion-mri.googlecode.com/svn/trunk/Python/lsqnonneg.py`

7 4C-seq

The Circular Chromosome Conformation Capture and sequencing (4C-seq) is derived from an earlier technique that used hybridization to microarrays (Simonis *et al.*, 2006). It aims at identifying long-range 3-dimensional interaction partners along the genome by mapping physical interactions between pre-selected bait loci and the rest of the genome.

The analysis pipeline implemented in HTSstation is based on the method used in the study (Noordermeer *et al.*, 2011), and described in (Gheldof *et al.*, 2012) (see Figure 1). It goes through several steps to transform a list of raw reads into a genome-wide pattern of interaction partners:

- **(Virtual) Library Preparation:** For each reference genome, a list of all potential restriction fragments is compiled by searching all occurrences of the first and second enzyme recognition sites (cutters). A restriction fragment is delimited by two consecutive occurrences of the first cutter. A valid fragment contains at least one occurrence of the second cutter at a sufficient distance from the first cutter. For species available in Repbase (mouse, human and drosophila), we quantify the overlap with repeats of each fragment edge using BEDtools and possibly mask them (see (Gheldof *et al.*, 2012) for more information about the rationale of this library).

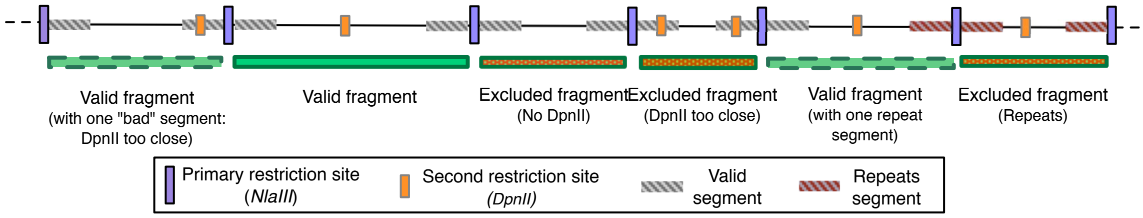

Figure 4: Different configurations of the restriction fragments

- **Counts per restriction fragments:** As sequencing reads are generated from either sides of every fragment, reads mapping to each end are counted (from either strand without PCR duplicates correction). Scores obtained at the two fragment ends are summed to assign a single value per fragment. If one side of the fragment intersects a repetitive region, this value is ignored and the other side is doubled. If both sides intersect repeats, the fragment is removed. The final value is normalized to the total of all fragment values. Finally, only valid fragments are reported.

- **Data smoothing:** Fragment scores are smoothed with a moving average calculated as follows (for fragment f_i in a window of size $2n + 1$):

$$s(f_i) = \frac{1}{2n+1} \sum_{k=i-n}^{i+n} s_0(f_k) \; .$$

- **4C profile analysis:** Typical 4C-seq profiles show very high fragment scores in the close neighbourhood of the viewpoint (the local saturated domain, see (Noordermeer *et al.*, 2011)). As this effect is mainly due to genomic proximity with the viewpoint and therefore does not constitute meaningful long range interactions, we have included an option to compensate this local effect by fitting a power-law model (see (Lieberman-Aiden *et al.*, 2009)). Corrected scores are obtained as follows: let the $s(f)$ and $d(f)$ be the score and distance to the bait for fragment f. The distance is measured from the middle of the fragment to the middle of the bait region. We bin fragments on a logarithmic scale:

$$d_{\text{bin}}(f) = 2^{\text{round}\left(\log_2 d(f)\right)} \; .$$

Within each bin we compute the average score:

$$s_{\text{bin}}(f) \;=\; \text{mean}\Big\{ s(g) \;:\; d_{\text{bin}}(f) \;=\; d_{\text{bin}}(g) \Big\} \;.$$

The corrected score $s'(f)$ is the ratio of the actual score to a fit by a power law of exponent -1:

$$s'(f) \;=\; \frac{s(f) \cdot d(f)}{d(f) + \exp\Big(\langle \log s_{\text{bin}}(f) \rangle + \langle \log d_{\text{bin}}(f) \rangle\Big)} \;.$$

We take into account potential asymmetries by treating up- and downstream profiles independently.

As mentioned in (Gheldof *et al.*, 2012), a 4C-seq data analysis seeks clusters of over-represented restriction fragments, rather than isolated high-frequency fragments. To identify such clusters, we implemented an approach based on (de Wit *et al.*, 2008). This method combines the notion of domainograms with a partitioning algorithm to infer the optimal discrete domains of enrichment.

8 SNP analysis

Analysis of polymorphisms is a post-processing of genome alignments and takes BAM files as input (see Figure 1). The SAMtools pileup functionality (Li *et al.*, 2009) is used to summarize variants (single base differences between reads and the genome at the aligned positions) which are then processed with a Python script to filter out SNPs with read coverage below T_{cover} and proportion of reads confirming a SNP below T_{snp}. The following default values are used depending on genome ploidy:

- T_{cover}: 10/allele,
- T_{snp}: 80% of an allele, at least 5 reads.

The output file contains a table listing all positions with a SNP detected in at least one of the processed samples, and the status of each sample at these positions.

The final step of this module consists in annotating SNPs. We provide SNP localization (exon, intron, intergenic) and in case of localization within a coding sequence we evaluate if the mutation is synonymous or non-synonymous.

References

Anders, S. and Huber, W. (2010). Differential expression analysis for sequence count data. *Genome Biology*, **11**(10), R106.

Blankenberg, D., Taylor, J., Schenck, I., He, J., Zhang, Y., Ghent, M., Veeraraghavan, N., Albert, I., Miller, W., Makova, K. D., Hardison, R. C., and Nekrutenko, A. (2007). A framework for collaborative analysis of ENCODE data: making large-scale analyses biologist-friendly. *Genome Research*, **17**(6), 960–4.

de Wit, E., Braunschweig, U., Greil, F., Bussemaker, H. J., and van Steensel, B. (2008). Global chromatin domain organization of the ¡italic¿drosophila¡/italic¿ genome. *PLoS Genet*, **4**(3), e1000045.

Gheldof, N., Leleu, M., Noordermeer, D., Rougemont, J., and Reymond, A. (2012). Detecting long-range chromatin interactions using the Chromosome Conformation Capture sequencing (4C-seq) method. In B. Deplancke and N. Gheldof, editors, *Gene Regulatory Networks: Methods and Protocols*, volume 786 of *Methods in Molecular Biology*, pages 211–225. Springer Verlag, New York, Berlin.

Giardine, B., Riemer, C., Hardison, R. C., Burhans, R., Elnitski, L., Shah, P., Zhang, Y., Blankenberg, D., Albert, I., Taylor, J., Miller, W., Kent, W. J., and Nekrutenko, A. (2005). Galaxy: a platform for interactive large-scale genome analysis. *Genome Research*, **15**(10), 1451–5.

Goldfarb, D. and Idnani, A. (1983). A numerically stable dual method for solving strictly convex quadratic programs. *Mathematical Programming*, **27**(1), 1–33.

Langmead, B., Trapnell, C., Pop, M., and Salzberg, S. (2009). Ultrafast and memory-efficient alignment of short DNA sequences to the human genome. *Genome Biology*, **10**(3), R25.

Lawson, C. L. and Hanson, R. J. (1974). *Solving least squares problems*. Prentice-Hall Inc., Englewood Cliffs, N.J.

Li, H., Handsaker, B., Wysoker, A., Fennell, T., Ruan, J., Homer, N., Marth, G., Abecasis, G. R., Durbin, R., and Subgroup, . G. P. D. P. (2009). The Sequence Alignment/Map format and SAMtools. *Bioinformatics*, **25**(16), 2078–2079.

Lieberman-Aiden, E., van Berkum, N. L., Williams, L., Imakaev, M., Ragoczy, T., Telling, A., Amit, I., Lajoie, B. R., Sabo, P. J., Dorschner, M. O., Sandstrom, R., Bernstein, B., Bender, M. A., Groudine, M., Gnirke, A., Stamatoyannopoulos, J., Mirny, L. A., Lander, E. S., and Dekker, J. (2009). Comprehensive mapping of long-range interactions reveals folding principles of the human genome. *Science*, **326**(5950), 289–93.

Noordermeer, D., Leleu, M., Splinter, E., Rougemont, J., De Laat, W., and Duboule, D. (2011). The dynamic architecture of hox gene clusters. *Science*, **334**(6053), 222–5.

Pepke, S., Wold, B., and Mortazavi, A. (2009). Computation for ChIP-seq and RNA-seq studies. *Nature methods*, **6**(11 Suppl), S22–32.

Rowe, A., Kalaitzopoulos, D., Osmond, M., Ghanem, M., and Guo, Y. (2003). The discovery net system for high throughput bioinformatics. *Bioinformatics*, **19 Suppl 1**, i225–31.

Rowe, H. M., Jakobsson, J., Mesnard, D., Rougemont, J., Reynard, S., Aktas, T., Maillard, P. V., Layard-Liesching, H., Verp, S., Marquis, J., Spitz, F., Constam, D. B., and Trono, D. (2010). KAP1 controls endogenous retroviruses in embryonic stem cells. *Nature*, **463**(7278), 237–240.

Simonis, M., Klous, P., Splinter, E., Moshkin, Y., Willemsen, R., de Wit, E., van Steensel, B., and de Laat, W. (2006). Nuclear organization of active and inactive chromatin domains uncovered by chromosome conformation capture-on-chip (4c). *Nat Gene*, **38**(11), 1348–54.

Slater, G. S. C. and Birney, E. (2005). Automated generation of heuristics for biological sequence comparison. *BMC Bioinformatics*, **6**, 31.

Zhang, Y., Liu, T., Meyer, C., Eeckhoute, J., Johnson, D., Bernstein, B., Nussbaum, C., Myers, R., Brown, M., Li, W., and Liu, X. (2008). Model-based analysis of ChIP-Seq (MACS). *Genome Biol*, **9**(9), R137.
