# Supplementary material for: HTSstation: A Web Application and Open-Access Libraries for High-Throughput Sequencing Data Analysis
Source: PLoS One. 2014 Jan 27;9(1):e85879. doi: 10.1371/journal.pone.0085879 (PMC3903476; doi:10.1371/journal.pone.0085879)
Supplement: File S2 — Pictorial description of a complete RNA-seq analysis process. A. Job description page for the mapping module. The first block defines the data sources and is structured according to experimental design, the second block is a general description usually common to successive jobs in the same analysis sequence, the third block sets module-specific options. B. Mapping results page, with links to the source files, to the results files, and to viewing tools. Notice the unique identifier at the top of the page. C. Processing further to the RNA-seq differential expression analysis involves importing the mapping results into the RNA-seq module using one or more job identifiers. D. RNA-seq results are presented in a similar way to panel B with additional viewing options (MA-plots). E. The interactive MA-plot provides a quick overview of differential expression between two conditions. (PDF) [file pone.0085879.s002.pdf]

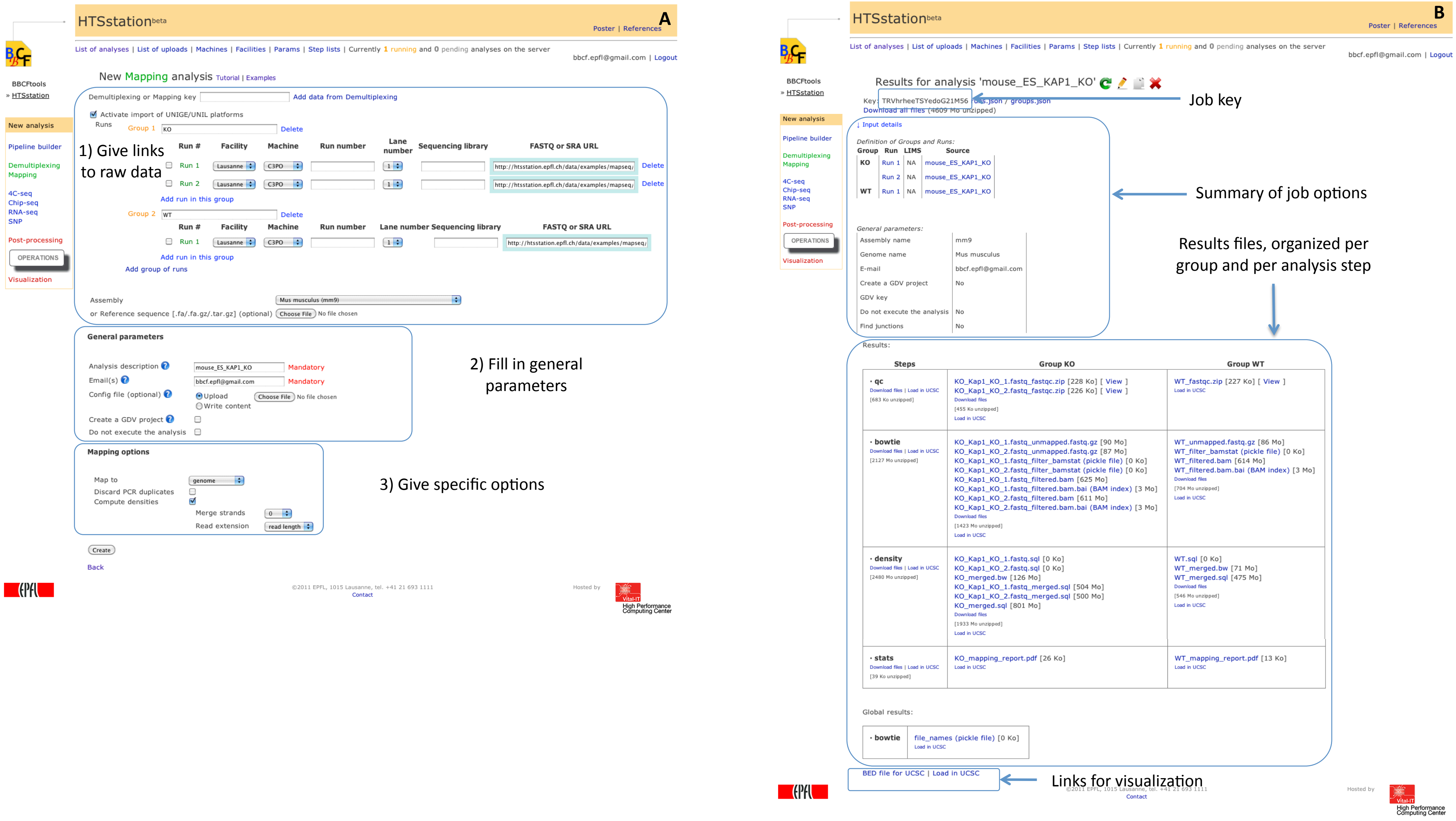
A
HTSstation beta
New Mapping analysis
1) Give links to raw data
General parameters
2) Fill in general parameters
Mapping options
3) Give specific options
B
HTSstation beta
Results for analysis 'mouse_ES_KAP1_KO'
Job key
Summary of job options
Results files, organized per group and per analysis step
Links for visualization

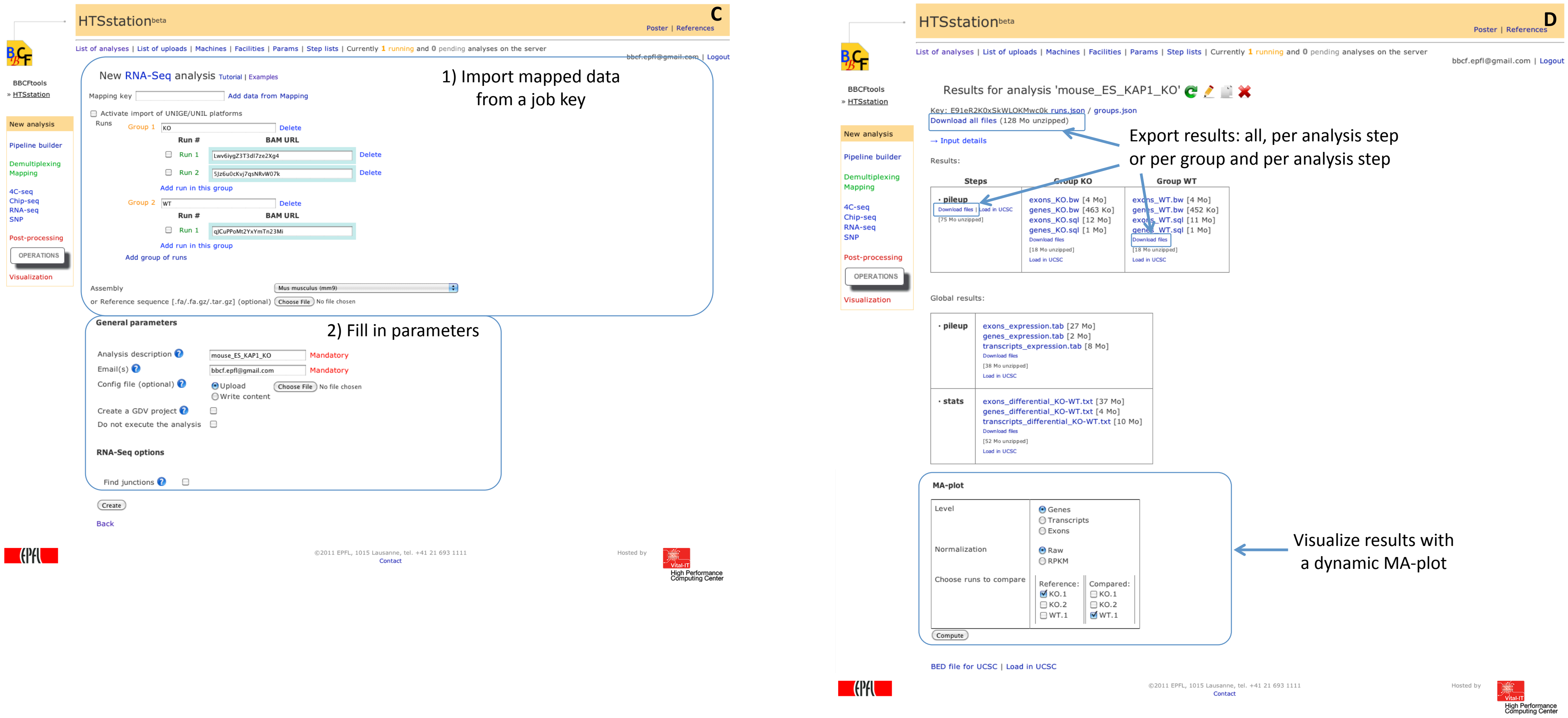

C
HTSstation beta
Poster | References
List of analyses | List of uploads | Machines | Facilities | Params | Step lists | Currently 1 running and 0 pending analyses on the server
New RNA-Seq analysis Tutorial | Examples
1) Import mapped data from a job key
Mapping key
Add data from Mapping
Activate import of UNIGE/UNIL platforms
Runs
Group 1 KO Delete
Run # BAM URL
Run 1 Delete
Run 2 Delete
Add run in this group
Group 2 WT Delete
Add group of runs
Assembly Mus musculus (mm9)
or Reference sequence [.fa/.fa.gz/.tar.gz] (optional) Choose File No file chosen
General parameters
2) Fill in parameters
Analysis description mouse_ES_KAP1_KO Mandatory
Email(s) bbcf.epfl@gmail.com Mandatory
Config file (optional) Upload Write content
Create a GDV project
Do not execute the analysis
RNA-Seq options
Find junctions
Create
Back
D
Results for analysis 'mouse_ES_KAP1_KO'
Download all files (128 Mo unzipped)
→ Input details
Results:
Steps Group KO Group WT
Export results: all, per analysis step or per group and per analysis step
Global results:
pileup
exons_expression.tab [27 Mo]
genes_expression.tab [2 Mo]
transcripts_expression.tab [8 Mo]
stats
exons_differential_KO-WT.txt [37 Mo]
genes_differential_KO-WT.txt [4 Mo]
transcripts_differential_KO-WT.txt [10 Mo]
MA-plot
Level Genes Transcripts Exons
Normalization Raw RPKM
Choose runs to compare Reference: KO.1 KO.2 WT.1 Compared: KO.1 KO.2 WT.1
Compute
Visualize results with a dynamic MA-plot
BED file for UCSC | Load in UCSC

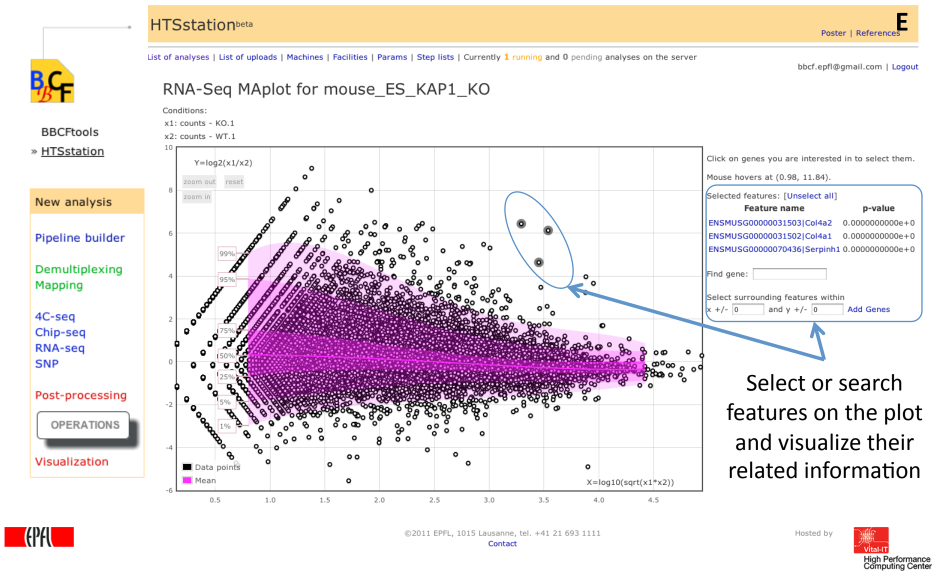
E
HTSstation beta
Poster | References
List of analyses | List of uploads | Machines | Facilities | Params | Step lists | Currently 1 running and 0 pending analyses on the server
bbcf.epfl@gmail.com | Logout
BBCFtools
» HTSstation
New analysis
Pipeline builder
Demultiplexing
Mapping
4C-seq
Chip-seq
RNA-seq
SNP
Post-processing
OPERATIONS
Visualization
RNA-Seq MAplot for mouse_ES_KAP1_KO
Conditions:
x1: counts - KO.1
x2: counts - WT.1
Y=log2(x1/x2)
zoom out
reset
zoom in
Data points
Mean
X=log10(sqrt(x1*x2))
Click on genes you are interested in to select them.
Mouse hovers at (0.98, 11.84).
Selected features: [Unselect all]
Feature name
p-value
ENSMUSG00000031503|Col4a2 0.0000000000e+0
ENSMUSG00000031502|Col4a1 0.0000000000e+0
ENSMUSG00000070436|Serpinh1 0.0000000000e+0
Find gene:
Select surrounding features within
x +/- 0 and y +/- 0 Add Genes
Select or search features on the plot and visualize their related information
©2011 EPFL, 1015 Lausanne, tel. +41 21 693 1111
Contact
Hosted by
Vital-IT
High Performance Computing Center
